# The puzzling phylogeography of the haplochromine cichlid fish *Astatotilapia burtoni*

**DOI:** 10.1002/ece3.4092

**Published:** 2018-05-02

**Authors:** Gaëlle Pauquet, Walter Salzburger, Bernd Egger

**Affiliations:** ^1^ Zoological Institute University of Basel Basel Switzerland

**Keywords:** *Astatotilapia burtoni*, haplochromine cichlid, laboratory strain, Lake Tanganyika, phylogeography, RAD sequencing

## Abstract

*Astatotilapia burtoni* is a member of the “modern haplochromines,” the most species‐rich lineage within the family of cichlid fishes. Although the species has been in use as research model in various fields of research since almost seven decades, including developmental biology, neurobiology, genetics and genomics, and behavioral biology, little is known about its spatial distribution and phylogeography. Here, we examine the population structure and phylogeographic history of *A. burtoni* throughout its entire distribution range in the Lake Tanganyika basin. In addition, we include several *A. burtoni* laboratory strains to trace back their origin from wild populations. To this end, we reconstruct phylogenetic relationships based on sequences of the mitochondrial DNA (mtDNA) control region (d‐loop) as well as thousands of genomewide single nucleotide polymorphisms (SNPs) derived from restriction‐associated DNA sequencing. Our analyses reveal high population structure and deep divergence among several lineages, however, with discordant nuclear and mtDNA phylogenetic inferences. Whereas the SNP‐based phylogenetic hypothesis uncovers an unexpectedly deep split in *A. burtoni*, separating the populations in the southern part of the Lake Tanganyika basin from those in the northern part, analyses of the mtDNA control region suggest deep divergence between populations from the southwestern shoreline and populations from the northern and southeastern shorelines of Lake Tanganyika. This phylogeographic pattern and mitochondrial haplotype sharing between populations from the very North and the very South of Lake Tanganyika can only partly be explained by introgression linked to lake‐level fluctuations leading to past contact zones between otherwise isolated populations and large‐scale migration events.

## 1 INTRODUCTION

With an estimated number of 3,000–5,000 species, the Cichlidae represent what is perhaps the most species‐rich family of teleost fishes (Turner, Seehausen, Knight, Allender, & Robinson, 2001). Throughout their range, but particularly in the East African Great Lakes, cichlid fishes have repeatedly undergone adaptive radiation and explosive speciation and are thus well‐known model systems to study these processes (see, e.g., Kocher, 2004; Salzburger, 2009; Santos & Salzburger, 2012). Within the Cichlidae, the “modern haplochromines” (sensu Salzburger, Mack, Verheyen, & Meyer, 2005) represent the most species‐rich lineage. They supposedly originated in the area of Lake Tanganyika and subsequently colonized other water bodies in Africa, thereby seeding the adaptive radiations of lakes Malawi and Victoria, among others (Koblmuller, Sefc, & Sturmbauer, 2008; Salzburger et al., 2005; Verheyen, Salzburger, Snoeks, & Meyer, 2003). It is believed that habitat generalist species were the ones who colonized lakes via a series of temporal river connections, thus transporting genetic polymorphisms across large areas in East Africa (Loh et al., 2013; Malinsky et al., 2015; Salzburger et al., 2005).

**Figure 1:**
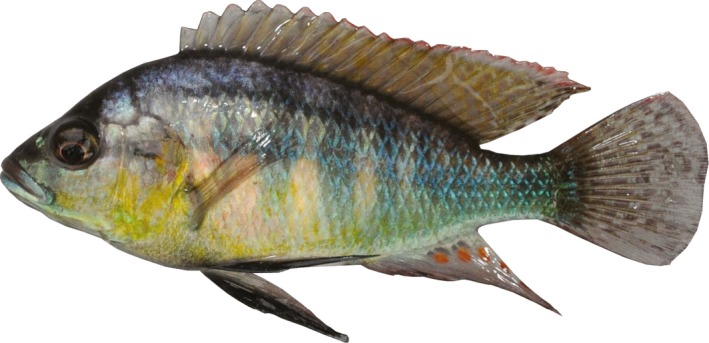
Photograph of a male *Astatotilapia burtoni* from Lake Cohoha, Burundi

*Astatotilapia burtoni* (Günther, 1893; Figure 1), which occurs both within Lake Tanganyika proper and in rivers belonging to the Lake Tanganyika drainage system, is such a generalist haplochromine cichlid (De Vos, Snoeks, & Van Den Audernaerde, 2001; Fernald & Hirata, 1977b; Kullander & Roberts, 2011). Phylogenetically, *A. burtoni* is nested with the “modern haplochromines” as one of several sister lineages to the Lake Malawi assemblage and the Lake Victoria region superflock (Meyer, Matschiner, & Salzburger, 2015; Salzburger et al., 2005). The species is among the five African cichlids to have a complete reference genome sequence (Brawand et al., 2014) and constitutes one of the most important cichlid model species in various fields of research, including developmental biology, neurobiology, genetics and genomics, and behavioral biology (see, e.g., Baldo, Santos, & Salzburger, 2011; Diepeveen, Roth, & Salzburger, 2013; Dijkstra et al., 2017; Egger, Roesti, Bohne, Roth, & Salzburger, 2017; Hofmann, 2003; Juntti et al., 2016; Lang et al., 2006; Robison et al., 2001; Salzburger et al., 2008; Santos et al., 2014; Theis, Salzburger, & Egger, 2012; Wickler, 1962).

Despite the species’ application as research model since almost seven decades (e.g., Leong, 1969; Wickler, 1962), little is known about the ecology and behavior of this species in nature, and there is a lack of knowledge on its spatial distribution and phylogeography. Such information is crucial, however, to understand the biology of a species and to interpret laboratory‐based experimental results. Moreover, the geographic origin and genetic relationships of *A. burtoni* laboratory strains used in different studies are in many cases not reported or unknown.

Previous work, focussing on the adaptive divergence of *A. burtoni* from lake and stream habitats, already reported high levels of genetic diversity in mitochondrial DNA (mtDNA) and microsatellite markers among populations examined from the southern part of Lake Tanganyika, as well as a deep split between populations from the eastern shoreline, the western shoreline and the headwaters of the Lufubu River (Theis, Ronco, Indermaur, Salzburger, & Egger, 2014). The observed distribution of the main mtDNA haplotype lineages was interpreted to reflect past lake‐level oscillations (Theis et al., 2014). Such fluctuations in the lake level, caused by variation in hydrology through time (Cohen, Lezzar, Tiercelin, & Soreghan, 1997; McGlue et al., 2010; Scholz et al., 2007), have previously been documented to affect population dynamics in rock‐dwelling, littoral cichlid species from lakes Tanganyika (Baric, Salzburger, & Sturmbauer, 2003; Koblmüller et al., 2011; Sturmbauer, Baric, Salzburger, Rüber, & Verheyen, 2001) and Malawi (Genner, Knight, Haesler, & Turner, 2010). In a follow‐up study based on single nucleotide polymorphisms (SNPs) derived from genomic DNA (via restriction site‐associated DNA sequencing; RADseq), we confirmed a deep divergence in *A. burtoni* populations in the South of Lake Tanganyika, in this case, however, between the Lufubu River and all remaining populations including the fish sampled at the estuary of the Lufubu River (Egger et al., 2017). Taken together, previous studies not only cover a small fraction of the distribution range of *A. burtoni*, but revealed somewhat conflicting results with respect to population structure in this species.

In this study, we examine the population structure and phylogeographic history of *A. burtoni* throughout its entire distribution range in the Lake Tanganyika basin. To this end, we extend our population sample to now include specimens collected within the lake and in inflowing rivers along the entire shoreline of Lake Tanganyika and reconstruct phylogenetic relationships based on sequences of the mtDNA control region (d‐loop) as well as thousands of genomewide SNPs derived from RADseq. We then explore the population structure via nearest neighbor haplotype co‐ancestry analyses. Finally, by including samples from different laboratory strains in phylogenetic and population genetic analyses, we trace back their origins from wild populations.

## 2 MATERIAL AND METHODS

### 2.1 Study sites, sampling, and DNA extraction

**Figure 2:**
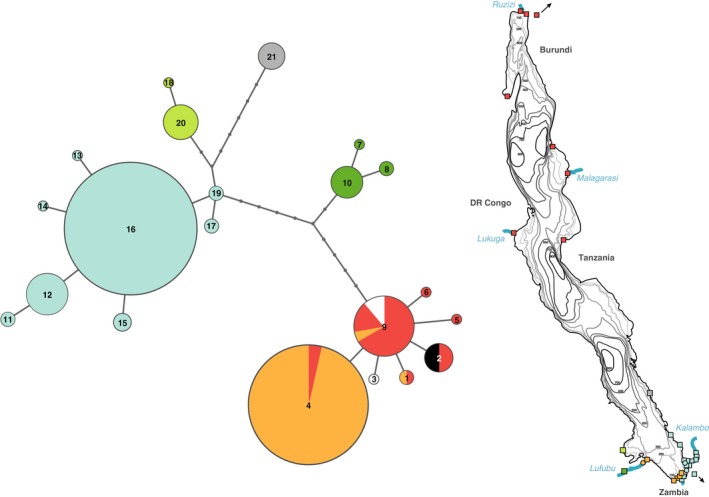
Haplotype genealogy based on sequences of the mtDNA control region showing the 21 haplotypes and the deep split between the northern/southwestern lineage and the southeastern lineage. Colors represent geographic regions as indicated on the map of Lake Tanganyika (red: northern populations, orange: southwestern populations, blue: southeastern populations, dark green: Lufubu stream 2 (LF2), light green: Ndole Bay (NDB), gray: Ninde (NIN); see Figure 3 for names of sampling locations). Laboratory strains are indicated in white (HHL, haplotype 9) and black (LAB, haplotype 2)

**Figure 3:**
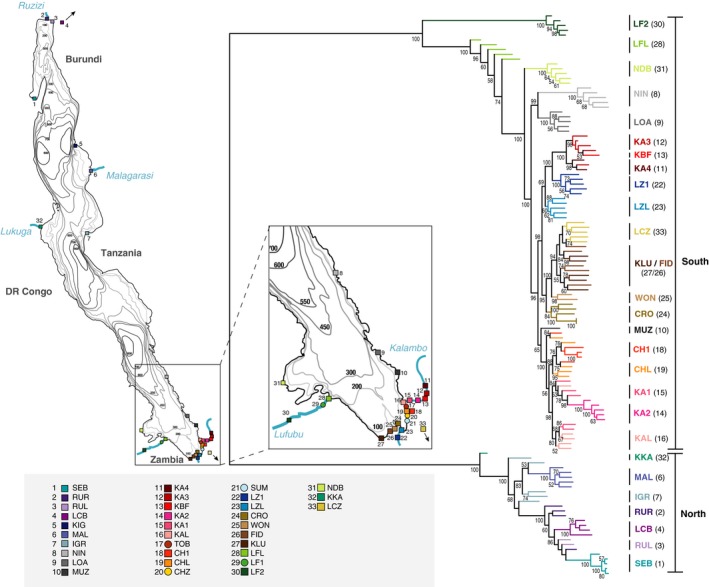
Map of LT showing sampling locations and nuclear phylogeny based on RADseq. Populations sampled at the shorelines of Lake Tanganyika (*n* = 31), Lake Cohoha (LCB,* n* = 1), and Lake Chila (LCZ,* n* = 1; full names of localities are given in Table S1). The unrooted maximum‐likelihood tree based on 19,037 SNPs and 117 individuals shows a deep split between northern and southern lineages. Colors in the phylogeny correspond to the colors on the map; bootstrap support of nodes is given in per cent. Note that samples from locations 17, 20, 21 and 29 were included for mtDNA analysis only

Sampling was carried out between February 2010 and November 2015 in the Zambian, Tanzanian, and Burundian parts of Lake Tanganyika and inflowing rivers, as well as in Lake Cohoha (Burundi) and Lake Chila (Zambia) (Figures 2 and 3). All specimens were caught using minnow traps or hook and line, with the approval of the Department of Fisheries Republic of Zambia (study permits 001994 and 003376), the Tanzanian Commission for Science and Technology (COSTECH; permit no. 2015‐171‐NA‐2015‐96), and the University of Burundi and the Ministry of Water and Environment, Republic of Burundi (Nr. 2014/R991). Due to our long‐term collaboration with the Department of Fisheries Republic of Zambia and more frequent sampling expeditions to the southern part of the lake, there is a better sampling coverage of the Southern basin as compared to the Central and Northern basins. Fish handling at the University of Basel was covered by permit no. 2317 issued by the cantonal veterinary office, Basel. Samples from Kalemie, Democratic Republic of Congo, and Kigoma, Tanzania, were collected and kindly provided by M. Van Steenberge (University of Leuven, Belgium); samples from Sebele, Democratic Republic of Congo, were collected and kindly provided by Y. Fermon (Association Aimara, France); and a specimen from the Kalambo River just below the Kalambo Falls was collected and kindly provided by F. Schedel (The Bavarian State Collection of Zoology, Munich, Germany). We further included specimens from strains of *A. burtoni* used in research laboratories, one bred at the University of Texas at Austin, USA, provided by H. Hofman/S. Renn and established by R. Fernald from wild collections from the Ruzizi area in Burundi (collected in 1975; Fernald & Hirata, 1977b), and one bred at our own laboratory and derived from a laboratory stock established by O. Seehausen. H. Hofman/S. Renn provided an additional set of five wild‐caught samples. In total, we gathered samples from 33 locations and two laboratory strains (see Table S2 for details). All fish collected by the authors of this study were anesthetized with clove oil prior to handling; all specimens were photographed, sized, weighted, and sexed, and a fin clip was taken as DNA sample and stored in 96% ethanol. DNA extraction was performed with the E.Z.N.A.^®^ Tissue DNA Kit (Omega Bio‐tek^®^) according to the manufacturer's protocol.

### 2.2 Mitochondrial control region sequencing and analysis

Amplification of a 374‐bp fragment of the mitochondrial control region (d‐loop) was conducted using published primers (L‐Pro‐F and TDK‐D; Kocher et al., 1989; Salzburger, Meyer, Baric, Verheyen, & Sturmbauer, 2002) and following a published protocol (Theis et al., 2014). PCR products were purified with Exo‐SAP‐IT (USB) and Sanger‐sequenced on an ABI 3130*xl* genetic analyzer using the BigDye Terminator v3.1 Cycle Sequencing Kit (Applied Biosystems). Sequences obtained in this study (*n* = 62; available at GenBank under the accession numbers MG987216–MG987279) were supplemented with available data from previous work (Salzburger et al., 2005; Theis et al., 2014; Verheyen et al., 2003), leading to a data set containing mtDNA sequence information of 428 specimens. DNA sequences were aligned using CODONCODE ALIGNER (v.3.5; CodonCode Corporation) and MAFFT (http://www.ebi.ac.uk/Tools/msa/mafft/). fabox (Villesen, 2007) was applied to collapse sequences into haplotypes. We then used FITCHI (Matschiner, 2016) to construct an unrooted haplotype genealogy following the method described in Salzburger, Ewing, and von Haeseler (2011) with increased node sizes relative to the branches and population‐specific haplotypes (‐m 2 and –p option).

### 2.3 RAD library preparation and sequencing

For RAD sequencing, we selected one to five individuals per sampling location and obtained a total of 150 individuals from 29 locations and including both laboratory strains. Libraries were prepared according to the protocol described in Roesti, Hendry, Salzburger, and Berner (2012). In short, a DNA concentration of 20 ng/μl was used for library preparation allowing for a deviation of ±1 ng/μl. Genomic DNA was digested using the restriction enzyme *Sbf1* and 5‐mer barcoded followed by subsequent P1 adapter ligation. After barcoding, 38–40 individuals were pooled per RAD library. The DNA was sheared to an average size of approx. 500 bp using a Bioruptor UCD‐300 and cleanup was performed using MinElute™PCR purification kit (Qiagen). The libraries were size selected on a gel before P2 adapter ligation was performed. The final enrichment PCR was split into six separate reactions per library to avoid amplification bias. The readily prepared libraries were single‐end sequenced in 100–200 cycles on an Illumina HiSeq 2000 platform at the Genomics Facility Basel jointly operated by ETH Zurich Department of Biosystems Science and Engineering (D‐BSSE) and the University of Basel. Illumina reads are available from the Sequence Read Archive (SRA) at NCBI under the accession numbers SRX2967972 **–** SRX2968211 (SRA Study Number: SRP110734) and SRX3733973–SRX3734072 (SRA Study Number: SRP 133290).

### 2.4 RAD data processing

The obtained RADseq reads were quality filtered, sorted according to barcode, and aligned to the *A. burtoni* reference genome (release Broad HapBur1.0, Brawand et al., 2014; using novoalign v2.08.03 (http://novocraft.com). The alignment score was set to 200 with a default gap‐opening penalty and a gap extend penalty of 15 accepted (parameters implemented for the alignment: ‐F STDFQ ‐t200 ‐g40 ‐×15 ‐oSAM ‐oFullNW –3Prime ‐rN ‐e10 –f) (see Egger et al., 2017). Mapping to the reference genome resulted in an average unique alignment success of 75.22% per individual. samtools, V.1.2 (Li et al., 2009) was used to convert the SAM file into BAM file format. Consensus genotypes at individual RAD loci were determined using a “genotype–haplotype” (sensu Nevado, Ramos‐Onsins, & Perez‐Enciso, 2014; calling approach introduced by Roesti, Kueng, Moser, & Berner, 2015). Diploids were called if the dominant haplotype occurred in at least 18 copies. A lighter representation of the dominant haplotype resulted in a haploid call, provided this haplotype was still present in more than two copies. For diploid loci, a RAD locus was considered heterozygous if the ratio of the dominant to the second most frequent haplotype was lower than 0.25. To avoid the unspecific alignment of sequence reads to several sites in the genome, we excluded RAD loci with a sequence coverage exceeding 3.5 times the expected mean coverage across all genomewide RAD loci (see Egger et al., 2017; Roesti et al., 2015).

Restriction site‐associated DNA tag processing was performed in R version 3.2.2, R Development Core Team (2012) using the sciCORE (http://scicore.unibas.ch/), the scientific computing core facility at University of Basel, with support from the Swiss Institute of Bioinformatics.

### 2.5 Phylogenetic analyses

After consensus genotype calling, SNP matrices were generated and converted to FASTA file format applying quality filtering. Only a single SNP with the highest minor allele frequency was allowed per RAD tag. SNPs with more than 20% missing data across all individuals were eliminated, and all individuals with more than 75% missing data dropped out likewise. We generated two different SNP matrices for phylogenetic analyses. The first dataset comprises *A. burtoni* samples from wild populations only (“SNP matrix wild”; 19,037 SNPs and 117 individuals). In the second SNP dataset, we included one specimen each of *Haplochromis paludinosus* (Greenwood, 1980), *Haplochromis falvijosephi* (Loret, 1883), and *Astatotilapia calliptera* (Günther, 1893) as outgroup taxa, plus the two laboratory strains and additional “wild” samples provided by the University of Texas (“SNP matrix lab_OG” comprising 20,892 SNPs and 132 Individuals; MAF = 0.01). We chose multiple riverine haplochromine species as outgroup taxa because of the uncertain sister‐group relationships among riverine haplochromines (see, e.g., Meyer et al., 2015; Salzburger et al., 2005). Note that in both matrices the samples from Kigoma, Tanzania (KIG (5)), dropped out due to poor quality.

Maximum‐likelihood trees were generated in R (version 3.2.2) using the phangorn package (Schliep, 2011). The appropriate phylogenetic model (GTR + G) was selected via jmodeltest (Posada, 2008), and a bootstrap analysis with 200 replicates was performed. The R package ape (Paradis, Claude, & Strimmer, 2004) was then used to visualize the phylogenetic tree.

### 2.6 Population genomic analyses

**Figure 4:**
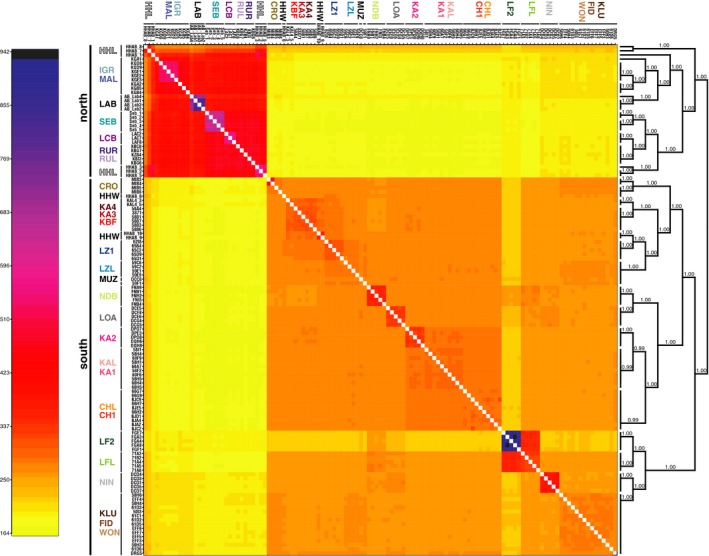
Clustered fine RAD structure co‐ancestry matrix. The highest levels of co‐ancestry are shared among individuals from the Lufubu stream population (LF2), indicated by black and blue colors. The lowest levels of co‐ancestry sharing are given among northern and southern populations, indicated by yellow coloration

We used the program fineRAdstructure (Malinsky, Trucchi, Lawson, & Falush, 2018) to infer population structure via shared ancestry among all *A. burtoni* individuals. The program is a modification of the fineSTRUCTURE package (Lawson, Hellenthal, Myers, & Falush, 2012) and has been specifically designed for RADseq data, as it does not require information about location of loci on chromosomes or phased haplotypes. The SNP matrix (including all samples except the outgroup specimens) was quality filtered to reduce the amount of missing data (by only allowing 10% missing data per SNP across all individuals and <40% missing data per individual), resulting in a matrix comprising 123 Individuals and 30,100 RAD loci. SNPs from the same RADtag were merged using a custom R script to generate the input file. The software RAdpainter, implemented in the fineRAdstructure package, was then applied to calculate the co‐ancestry matrix. As a next step, individuals were assigned to populations, with Markov Chain Monte Carlo simulations running for 100,000 replications, burn‐in = 100,000. Tree building was performed using default parameters. To visualize results, we used the R scripts fineRADstructurePlot.R and FinestructureLibrary.R (available at http://cichlid.gurdon.cam.ac.uk/fineRADstructure.html). After quality filtering and co‐ancestry matrix construction, almost all populations were still represented with at least one individual, except for KIG and KKA (Figure 4).

## 3 RESULTS

### 3.1 D‐loop haplotype genealogy

The d‐loop haplotype genealogy based on a 374‐bp fragment revealed the presence of 21 haplotypes and a deep split between the northern/southwestern lineages and the southeastern lineages (Figure 2). The northern/southwestern lineage comprises in total seven haplotypes, some of which are shared among northern and southwestern populations (haplotypes 1, 4, and 9). Eight haplotypes correspond to the southeastern populations (haplotypes 11, 12, 13, 14, 15, 16, 17, and 19). The Ndole Bay population (NDB; haplotypes 18 and 20) from the western shoreline in Zambia clustered with the populations from the southeastern shoreline. The Ninde population (NIN) from the Tanzanian shoreline represents a distinct haplotype (haplotype 21), but groups with the southeastern populations. Furthermore, the most upstream Lufubu population (LF2) represents a haplotype lineage (haplotypes 7, 8, and 10), quite distinct from either of the two major haplotype lineages. The laboratory strain samples all grouped with the northern/southwestern haplotypes (haplotypes 9 and 3: laboratory strain from the University of Texas; haplotype 2: laboratory strain from the University of Basel); the “wild” samples from the University of Texas (collected at Kalambo and Lunzua rivers) shared haplotype 17 with other samples from the southeastern lineage. The sequences from samples collected in the southern part of Lake Tanganyika resulted the same haplotype network topology as shown in fig. 1 (b) in Theis et al. (2014). GenBank sequences from Verheyen et al. (2003) shared haplotype 9 with samples from the north/southwest, whereas sequences from Salzburger et al. (2005) shared haplotype 2 with laboratory strain samples from the University of Basel.

### 3.2 Phylogenetic reconstruction based on RAD data

The maximum‐likelihood analyses for each of two datasets resulted in well‐resolved and congruent topologies (see Figure 3 for the topology with wild samples only and Figure S1 for the topology including laboratory strains and outgroups).

The phylogenetic reconstruction of the specimens collected from the wild (comprising 19,037 SNPs and 117 individuals) revealed a deep split between a northern clade (geographically ranging from the Ruzizi River (RUR (2)) to the Igalula River (IGR (7)) on the eastern shore and Kalemie (KKA (32)) on the western shore of Lake Tanganyika; and including *A. burtoni* from Lake Cohoha (LCB (4))) and a southern clade (ranging from Ninde (NIN (8)) to the Ndole Bay (NDB (31)); and including *A. burtoni* from Lake Chila (LCZ (33))) (Figure 3). Within the northern clade, populations from the northern basin of Lake Tanganyika (SEB (1), RUL (3), RUR (2)) were nested within populations from the lake's central basin (KKA (32), MAL (6), IGR (7)). The specimens from Lake Cohoha (LCB (4)) were resolved together with the Ruzizi specimens (RUL (3), RUR (2)). Within the southern clade, there was a deep split between the upstream Lufubu population (LF2 (30)) and the remaining samples, within which the Lufubu lake population (LFL (28)) branched off first, followed by the geographically nearby Ndole Bay (NDB (31)) fish. The remaining populations were grouped—largely in accordance with geography—into four more or less well‐defined clades formed by (1) the specimens from Ninde (NIN (8)) and Loasi (LOA (9)) from Southern Tanzania; (2) the fish from the Lunzua estuary and river (LZ1 (22), LZL (23)) and the upstream populations of the Kalambo river (KBF (13), KA3 (12), KA4 (11)); (3) the populations around Mpulungu in Zambia (i.e., KLU, FID, WON) including the population at Crocodile Island (CRO (24)) plus the fish from small lake Chila (LCZ (33)); and (4) the populations from the lower Kalambo river with its corresponding lake population (KAL (16), KA1 (15), KA2 (14)) and nearby Chitili creek (CHL (19), CH1 (18)).

The phylogenetic reconstruction including laboratory strains and three outgroup taxa (comprising 20,892 SNPs and 132 individuals) resulted in a highly similar topology as described above (Figure S1). The inclusion of outgroup taxa did not provide additional phylogenetic information. Both laboratory strains were resolved within the northern clade: The laboratory strain from the University of Basel grouped as sister clade to all populations from the central basin except the sample from Kalemie (KKA (32)), whereas the laboratory strain from the University of Texas formed a monophyletic sister clade to the northernmost samples (RUR (2), LCB (4), RUL (3), and SEB (1)). The “wild” specimens from the Hofmann laboratory grouped with samples from LZL (23) (HH_AB_wild6) and Ka3 (12) (HH_AB_wild9 and HH_AB_wild10).

### 3.3 RAD co‐ancestry matrix

The clustered co‐ancestry matrix with fineRAdstructure (Figure 4) confirmed the deep split between the northern and southern lineages, as both form distinct clusters. The northern populations showed a higher degree of shared ancestry compared to the southern populations. Within the southern populations, individuals from the (LF2) population displayed the highest levels of co‐ancestry, and there was a high degree of shared ancestry between the (LF2) population and its adjacent lake population (LFL). Substantial population structuring is evident from high levels of within‐population co‐ancestry in the north: LCB, MAL, SEB, and the south: KA2, NIN, NDB, LOA, and LAB. Both laboratory strains also revealed high levels of shared ancestry.

## 4 DISCUSSION

In this study, we surveyed the phylogeographic history of the haplochromine cichlid species *A. burtoni*, a habitat generalist occurring within Lake Tanganyika as well as in inflowing rivers, and tested for genetic substructuring in the natural populations of this widely used model species.

Our phylogenetic reconstructions based on roughly 20,000 SNP markers derived from RADseq provide an unprecedented resolution of the phylogenetic relationships among different *A. burtoni* populations across the entire distribution range of this species. The SNP‐based phylogenetic hypothesis uncovers an unexpectedly deep split in *A. burtoni*, separating the populations in the southern part of the Lake Tanganyika basin from those in the northern part (Figure 3). This deep divergence is in line with the observed high levels of shared ancestry among individuals within both the southern and the northern lineages and the very low levels of shared ancestry between these two clades (Figure 4).

Interestingly, in both the southern and the northern clades of *A. burtoni*, representatives of riverine populations occupy the most ancestral positions in the phylogeny. In a recent study examining the patterns of genome divergence between lake and river populations of *A. burtoni* in four river systems in the South of Lake Tanganyika (Egger et al., 2017), we found that the Lufubu River fish (LF2) are distinct from the remaining populations examined in that study. Moreover, it was shown that individuals from the Lufubu lake population (LFL) share similar levels of co‐ancestry with individuals from their own population as with specimens collected at LF2; however, whereas LFL individuals also share co‐ancestry with the other lake and stream populations in the area, this is not the case for individuals from LF2 (see fig. 2 in Egger et al., 2017; Figure 3 of this study). The inclusion of specimens from 15 additional sampling localities in the southern part of Lake Tanganyika did not change these findings (Figures 2 and 3), corroborating that the *A. burtoni* populations in the South of Lake Tanganyika were originally colonized from Lufubu River stocks. In the northern clade, the specimen from Kalemie (which is associated with the Lukuga River) occupies the most ancestral branches, suggesting that *A. burtoni* have colonized the northern part of Lake Tanganyika starting from the Lukuga River. The Lukuga River is the only intermittent outflow of Lake Tanganyika connecting the lake to the Congo drainage via the Lualaba River at periods of high lake‐level stands (Cohen et al., 1997; Coulter, 1991; Lezzar et al., 1996). *Astatotilapia burtoni* is known to occur in the Lukuga River as far as 100 km downstream of its outlet at Kalemie (Kullander & Roberts, 2011; Poll, 1956), but has not been found downstream of the Niemba Falls. At present times, there is no connection between the Lufubu River and the Congo drainage. However, a past connection enabling faunal exchange between the Lufubu headwaters and the Congo system during extreme flooding or river capture events has previously been proposed (Koblmuller, Katongo, Phiri, & Sturmbauer, 2012; Koch et al., 2007). It thus seems plausible that *A. burtoni* originated in the upper Congo/Lufubu area and spread from there via the Lukuga toward the central and northern part of the Lake Tanganyika basin and via the Lufubu toward the lakes’ southern end. Although we refrain from performing a molecular clock analysis for *A. burtoni* here due to the lack of reliable external calibration points, previous demographic analyses provide a hint toward the temporal framework for the evolution of *A. burtoni*. Our previous analyses revealed that the *A. burtoni* populations from Lufubu River (LF2) and from the lake site near the estuary of the Lufubu River (LFL) diverged between 161–213 ka (Egger et al., 2017). That the here reported split between the southern and northern clade of *A. burtoni* is much deeper than the split between LF2 and LFL (Figure 2) suggests that the two main clades in *A. burtoni* diverged much earlier than ~200 ka.

Interestingly, the clear‐cut separation between northern and southern populations of *A. burtoni* as revealed by the genomewide SNPs derived from RADseq is not evident in the mtDNA‐based haplotype genealogy. Instead, our analyses of sequences of the mtDNA control region revealed three major mitochondrial lineages in *A. burtoni*, which are geographically distributed in a different way (Figure 2): (1) One mtDNA haplotype lineage consists exclusively of the specimens collected from the upstream Lufubu population (LF2) (haplotypes 7, 8, 10; colored in green in Figure 2); (2) a second haplotype lineage comprises all individuals from the southeastern part of Lake Tanganyika collected between Loasi (LOA) and Kapata (LZL) plus the individuals collected at Ndole Bay at the southwestern shore (haplotypes 11–21); and (3) a haplotype lineage including all specimens from the northern populations plus the specimens collected in the South of Lake Tanganyika between the estuary of the Lufubu River (LFL, LF1) and Wonzye Point (WON)/Crocodile Island (CRO) (haplotypes 1–6, 9). Thus, there is one haplotype lineage with a quite restricted geographic distribution (1), whereas another one shows a more or less lakewide distribution (3), whereby its southern range of occurrence is flanked—at both the eastern and the western shores of Lake Tanganyika—by populations belonging to a third lineage (2). In the area of the Lufubu River, representatives of all three haplotype lineages meet in close geographic proximity. It is of note that there is not a single *A. burtoni* population in our sample in which we found mtDNA sequences belonging to two different major haplotype lineages.

That some of the southern populations show quite distinct mtDNA haplotypes has already been reported in a previous study (Theis et al., 2014) and interpreted as being due to an underwater ridge around Wonzye Point (WON)/Crocodile Island (CRO) that might have acted as migration barrier at lake‐level lowstands between the southeastern and southwestern populations. Surprisingly, the lakewide sampling of the present study revealed mtDNA haplotype sharing between populations from the very North and the very South of Lake Tanganyika, which are more than 600 km apart from each other. For example, the most common haplotype in the South (haplotype 4) has also been found in specimens from Bujumbura (RUL) and Lake Cohoha (LCB), suggesting a rather recent connection between these populations, at least of their females. Given the deep nuclear DNA (ncDNA) divergence between the northern and southern lineages, this pattern in mtDNA is difficult to explain. On the other hand, evidence is accumulating that the replacement of mtDNA across large geographic distances, without apparent signatures of nuclear genomic admixis is more common than previously thought (e.g., Good, Vanderpool, Keeble, & Bi, 2015; Melo‐Ferreira, Seixas, Cheng, Mills, & Alves, 2014; Nevado, Fazalova, Backeljau, Hanssens, & Verheyen, 2011; Tang, Liu, Yu, Liu, & Danley, 2012). More general, discordance between nuclear and mtDNA phylogenetic inferences is known from many freshwater fish taxa and attributed to their high propensity to hybridize (see Wallis et al., 2017). In particular, in stenotopic, littoral cichlids from Lake Tanganyika—such as *Eretmodus cyanosticus*,* Tropheus moorii* and *Variabilichromis moorii*—such mtDNA/ncDNA discordance patterns due to introgression/hybridization have been linked to lake‐level fluctuations leading to past contact zones between otherwise isolated populations and large‐scale migration events (Koblmüller et al., 2011; Nevado, Mautner, Sturmbauer, & Verheyen, 2013; Sefc, Baric, Salzburger, & Sturmbauer, 2007; Sturmbauer et al., 2001). In the genus *Tropheus*, for example, populations from opposite shorelines in the central and southern basin of Lake Tanganyika have been shown to share identical mtDNA haplotypes (Sturmbauer, Koblmuller, Sefc, & Duftner, 2005; Sturmbauer et al., 2001). It is thus possible that severe lake‐level drops in the past could also have enabled migration of *A. burtoni* across the western and eastern shorelines as well as across the Central/Northern basin at times when Lake Tanganyika was either split into three separate basins or these basins were only connected through swampy areas (four level drops were probably severe enough to separate the basins, ~390–360 ka; 290–260 ka; 190–170 ka; 135–70 ka; see Danley et al., 2012). However, it remains difficult to conceive how lake‐level fluctuations could have mediated mtDNA introgression between the northernmost and southernmost populations. Recent human‐induced faunal translocation, although apparently happening occasionally and locally (see below), seems a rather unlikely scenario to explain the across‐lake sharing of mtDNA haplotypes, given the relatively large geographic distribution of the haplotypes in question and diametrically opposite signature in ncDNA.

Our analyses revealed other puzzling results regarding the phylogeography of *A. burtoni*. For example, we had previously noticed that the populations in the Kalambo River are not monophyletic, as the specimens collected from a population upstream the ~220 m Kalambo Falls (KA3) turned out to cluster with the specimens from Lunzua River (Egger et al., 2017). The inclusion of an additional population sample from further upstream the Kalambo Falls (KA4) confirms this finding (Figure 2), suggesting past migration between the upper Kalambo and the Lunzua River via a past river connection, probably triggered by tectonic movements leading to river capture events (see Cohen et al., 2013; Delvaux, Kervyn, Vittori, Kajara, & Kilembe, 1998). Our previous work revealed that fish collected from the Kalambo River downstream the Kalambo Falls (KA1, KA2) and at a lake side near the river mouth (KAL) form a clade (Egger et al., 2017; Theis et al., 2014), which led us to suggest that the more downstream populations were seeded by lake fish and that the Kalambo Falls form a barrier to gene flow. The present study, however, contains a specimen collected from the pool just below the Kalambo Falls (KBF), which clusters with the upstream populations KA3 and KA4 in the SNP‐based phylogeny (Figure 3). This implies that at least one individual must have survived a drop of more than 220 m (alternatively, a mouthbrooding female might have fallen down and the incubated eggs or larvae survived the plunge).

This study is also the first to report a pure lake population of *A. burtoni* in Lake Tanganyika that has no direct access to a nearby river via a stretch of shoreline. *Astatotilapia burtoni* has previously been reported to occur in habitats such as marshy marginal ponds or lagoons, always in association with inflowing rivers (Fernald & Hirata, 1977a). Our own previous work has challenged this view in that we investigated many lake populations and showed that lake fish are phenotypically and ecologically distinct from river fish (Egger et al., 2017; Theis et al., 2014, 2017). At Crocodile Island (CRO), which is situated about 1.2 km away from the closest (southeastern) shoreline, *A. burtoni* are found in a water depth of 5–8 m, indicating that *A. burtoni* can survive and maintain populations in a proper lake habitat.

The SNP‐based phylogeny further indicates two likely cases of human‐mediated translocation of *A. burtoni* from Lake Tanganyika into other water bodies. The close genetic relationship between the Lake Chila population (LCZ) and the populations around Mpulungu (KLU, FID), as already discussed in Theis et al. (2014), is most likely due to recent translocation. Lake Chila, a small and shallow lake at Mbala, approximately 30 km south of Mpulungu, Zambia, has regularly been stocked in the past (see Theis et al., 2014). Similarly, the sister clade relationship between samples from Lake Cohoha (LCB) and the Ruzizi estuary (RUL) indicates human‐mediated translocation of *A. burtoni* from Lake Tanganyika into the Lake Cohoha system, about 135 km away from Lake Tanganyika. Note that Lake Cohoha is not connected to the Lake Tanganyika drainage but belongs to the Nile system, and native haplochromine cichlids in that area have previously been associated with the fauna of the Lake Victoria region (Verheyen et al., 2003). To our knowledge, *A. burtoni* was recorded in Lake Cohoha for the first time in 1993 (collectors: Snoeks, Notenbaert & Vanlishout, MRAC, Trevuren). According to a FAO report from 1991 (FAO 1991), several cichlid species have been introduced into Lake Cohoha: *Tilapia rendalli* (now *Coptodon rendalli*), *Sarotherodon niloticus* (now *Oreochromis niloticus*), *S. macrochir* (now *O. macrochir*), and *Astatoreochromis alluaudi*. In this report, *A. burtoni* is not mentioned; however, an accidental introduction of the species, for example, in the course of stocking Lake Cohoha with *O. niloticus*, seems to be way more likely than natural dispersal.

Finally, the inclusion of two laboratory strains in the phylogenetic reconstruction revealed that both strains originally stem from the northern clade of *A. burtoni* (Figure S1). The laboratory strain from the University of Texas (HHL) grouped as sister clade to samples from the northern basin, which is in line with the original sampling site in the Ruzizi area. The origin of the Basel laboratory strain (LAB) is less clear, as it forms the sister clade to all samples from the northern and central basins (except the more basal Kalemie [KKA] sample). Since several decades, *A. burtoni* is a laboratory model for various research fields such as developmental biology, neurobiology, genetics and genomics, and behavioral biology (see, e.g., Baldo et al., 2011; Diepeveen et al., 2013; Dijkstra et al., 2017; Egger et al., 2017; Hofmann, 2003; Juntti et al., 2016; Lang et al., 2006; Robison et al., 2001; Salzburger et al., 2008; Santos et al., 2014; Theis et al., 2012; Wickler, 1962). Given the high population structure and deep divergence among several clades in *A. burtoni*, different populations and/or laboratory strains might also vary with regard to the trait(s) under study. In two recent studies dealing with the genomics of sex determination in *A. burtoni*, Böhne et al. (2016) inferred a XX/XY system located on LG5 for the laboratory strain of the University of Basel (LAB), and a XX/XY system at LG18 for a wild population from the southern lineage (KAL). Roberts et al. (2016), using a laboratory strain that is very likely from the same source population as the one from the University of Texas (HHL), also identified a XX/XY system on LG5 but an additional ZZ/ZW on LG13. Behavioral differences between the HH laboratory strain and southern populations (LZL and KA3) were observed in a study on maternal care (Renn et al., 2009). Hence, we deem it highly relevant to report which natural population or laboratory strain was used in publications in the future.

## CONFLICT OF INTEREST

None declared.

## AUTHOR CONTRIBUTIONS

W.S. and B.E. designed the research; G.P. and B.E. performed the wet laboratory work and analyzed the data; G.P., W.S., and B.E. wrote the manuscript.

## DATA ACCESSIBILITY

Raw RAD sequencing reads generated for this study are available from the Sequence Read Archive (SRA) at NCBI under the accession numbers SRX3733973–SRX3734072 (SRA Study Number: SRP 133290). Mitochondrial D‐loop sequences obtained in this study are available at GenBank under the accession numbers MG987216–MG987279.
