# Supplementary material for: The puzzling phylogeography of the haplochromine cichlid fish Astatotilapia burtoni
Source: Ecol Evol. 2018 May 2;8(11):5637–48. doi: 10.1002/ece3.4092 (PMC6010872; doi:10.1002/ece3.4092)
Supplement: Supplementary file 2 [file ECE3-8-5637-s002.pdf]

Species / *A. burtoni* population	Sample ID	D-loop	RAD	Origin	Latitude	Longitude	Reference: D-loop	Reference: RAD
Astatotilapia calliptera	A.cal2		x	-	-	-		
Astatotilapia flavijosephi	LJD2		x	-	-	-		
Bujumbura, Ruzizi lake (RUL)	AB_1	x		Burundi	3°21'27.42"S	29°17'3.18"E		
Bujumbura, Ruzizi lake (RUL)	AB_4	x		Burundi				
Bujumbura, Ruzizi lake (RUL)	IHA1	x		Burundi				
Bujumbura, Ruzizi lake (RUL)	IHA5	x		Burundi				
Bujumbura, Ruzizi lake (RUL)	IHA6	x		Burundi				
Bujumbura, Ruzizi lake (RUL)	IHA8	x		Burundi				
Bujumbura, Ruzizi lake (RUL)	KZB4	x	x	Burundi				
Bujumbura, Ruzizi lake (RUL)	KBH9	x	x	Burundi				
Bujumbura, Ruzizi lake (RUL)	KBI2	x	x	Burundi				
Bujumbura, Ruzizi lake (RUL)	KBI3	x	x	Burundi				
Bujumbura, Ruzizi lake (RUL)	KBH9		x	Burundi				
Chisanza (CHZ)	1783	x		Zambia	8°39'57.39"S	31°11'41.26"E	Theis et al. 2014	
Chisanza (CHZ)	1785	x		Zambia			Theis et al. 2014	
Chisanza (CHZ)	1786	x		Zambia			Theis et al. 2014	
Chisanza (CHZ)	1788	x		Zambia			Theis et al. 2014	
Chisanza (CHZ)	1790	x		Zambia			Theis et al. 2014	
Chisanza (CHZ)	1791	x		Zambia			Theis et al. 2014	
Chisanza (CHZ)	1792	x		Zambia			Theis et al. 2014	
Chisanza (CHZ)	1793	x		Zambia			Theis et al. 2014	
Chisanza (CHZ)	1794	x		Zambia			Theis et al. 2014	
Chitili creek (CH1)	46F2	x		Zambia	8°38'16.91"S	31°12'4.02"E	Theis et al. 2014	
Chitili creek (CH1)	46F3	x		Zambia			Theis et al. 2014	
Chitili creek (CH1)	46F4	x		Zambia			Theis et al. 2014	
Chitili creek (CH1)	46F5	x		Zambia			Theis et al. 2014	
Chitili creek (CH1)	46F6	x		Zambia			Theis et al. 2014	
Chitili creek (CH1)	46F7	x		Zambia			Theis et al. 2014	
Chitili creek (CH1)	46F8	x		Zambia			Theis et al. 2014	
Chitili creek (CH1)	BJA1	x		Zambia			Theis et al. 2014	
Chitili creek (CH1)	BJA2	x	x	Zambia			Theis et al. 2014	Egger et al. 2017
Chitili creek (CH1)	BJA3	x		Zambia			Theis et al. 2014	
Chitili creek (CH1)	BJA4	x	x	Zambia			Theis et al. 2014	
Chitili creek (CH1)	BJA5	x		Zambia			Theis et al. 2014	Egger et al. 2017
Chitili creek (CH1)	BJA6	x		Zambia			Theis et al. 2014	
Chitili creek (CH1)	BJA7	x		Zambia			Theis et al. 2014	
Chitili creek (CH1)	BJA8	x		Zambia			Theis et al. 2014	
Chitili creek (CH1)	BJA9	x		Zambia			Theis et al. 2014	
Chitili creek (CH1)	BJB1	x		Zambia			Theis et al. 2014	
Chitili creek (CH1)	BJC2		x	Zambia				Egger et al. 2017
Chitili creek (CH1)	BJC9		x	Zambia				Egger et al. 2017
Chitili creek (CH1)	BJD1		x	Zambia				Egger et al. 2017
Chitili lake (CHL)	66G7	x	x	Zambia	8°38'18.42"S	31°11'55.34"E	Theis et al. 2014	Egger et al. 2017
Chitili lake (CHL)	66G8	x		Zambia			Theis et al. 2014	
Chitili lake (CHL)	66G9	x	x	Zambia			Theis et al. 2014	Egger et al. 2017
Chitili lake (CHL)	66H1	x		Zambia			Theis et al. 2014	
Chitili lake (CHL)	66H2	x	x	Zambia			Theis et al. 2014	Egger et al. 2017
Chitili lake (CHL)	66H3	x		Zambia			Theis et al. 2014	
Chitili lake (CHL)	66H4	x		Zambia			Theis et al. 2014	
Chitili lake (CHL)	66H5	x		Zambia			Theis et al. 2014	
Chitili lake (CHL)	66H6	x		Zambia			Theis et al. 2014	
Chitili lake (CHL)	66H7	x	x	Zambia			Theis et al. 2014	Egger et al. 2017
Chitili lake (CHL)	BJE5		x	Zambia				Egger et al. 2017
Crocodile Island (CRO)	MIB3	x	x	Zambia	8°43'19.26"S	31° 7'19.42"E		
Crocodile Island (CRO)	MIB4	x	x	Zambia				
Crocodile Island (CRO)	MIB5	x	x	Zambia				
Crocodile Island (CRO)	MIB6	x	x	Zambia				
Crocodile Island (CRO)	MIC2	x	x	Zambia				
Fisheries Department (FID)	50G8	x		Zambia	8°45'58.52"S	31°6'23.99"E	Theis et al. 2014	
Fisheries Department (FID)	50G9	x		Zambia			Theis et al. 2014	
Fisheries Department (FID)	50H1	x		Zambia			Theis et al. 2014	
Fisheries Department (FID)	50H2	x		Zambia			Theis et al. 2014	
Fisheries Department (FID)	50H3	x	x	Zambia			Theis et al. 2014	
Fisheries Department (FID)	50H4	x	x	Zambia			Theis et al. 2014	
Fisheries Department (FID)	50H5	x	x	Zambia			Theis et al. 2014	
Fisheries Department (FID)	50H6	x	x	Zambia			Theis et al. 2014	
Fisheries Department (FID)	50H7	x		Zambia			Theis et al. 2014	
Fisheries Department (FID)	50H8	x		Zambia			Theis et al. 2014	
Fisheries Department (FID)	50H9	x		Zambia			Theis et al. 2014	
Fisheries Department (FID)	50I1	x		Zambia			Theis et al. 2014	
Fisheries Department (FID)	50I3	x	x	Zambia			Theis et al. 2014	
Fisheries Department (FID)	50I4	x		Zambia			Theis et al. 2014	
Fisheries Department (FID)	50I5	x		Zambia			Theis et al. 2014	
Fisheries Department (FID)	50I6	x		Zambia			Theis et al. 2014	
Fisheries Department (FID)	50I7	x		Zambia			Theis et al. 2014	
Fisheries Department (FID)	50I8	x		Zambia			Theis et al. 2014	
Fisheries Department (FID)	50I9	x		Zambia			Theis et al. 2014	
Fisheries Department (FID)	61A2_1	x		Zambia			Theis et al. 2014	
Fisheries Department (FID)	61A2_2	x		Zambia			Theis et al. 2014	
Fisheries Department (FID)	61A2_3	x		Zambia			Theis et al. 2014	
Fisheries Department (FID)	61A2_4	x		Zambia			Theis et al. 2014	
Fisheries Department (FID)	61A2_5	x		Zambia			Theis et al. 2014	
Haplochromis paludinosus	KYG1		x	-	-	-		
H. Hofmann Lab strain (HHL)	HHAB_1	x	x	Lab				

H. Hofmann Lab strain (HHL)	HHAB_2	x	x	Lab				
H. Hofmann Lab strain (HHL)	HHAB_3	x	x	Lab				
H. Hofmann Lab strain (HHL)	HHAB_4	x	x	Lab				
H. Hofmann Lab strain (HHL)	HHAB_5	x	x	Lab				
H.Hofmann wild (HHW)	HHAB_6	x	x	Zambia				
H.Hofmann wild (HHW)	HHAB_7	x	x	Zambia				
H.Hofmann wild (HHW)	HHAB_8	x	x	Zambia				
H.Hofmann wild (HHW)	HHAB_9	x	x	Zambia				
H.Hofmann wild (HHW)	HHAB_10	x	x	Zambia				
Igalula River Delta (IGR)	KGA1	x	x	Tanzania	05° 57' 09'' S	29° 52' 38'' E		
Igalula River Delta (IGR)	KGA2	x	x	Tanzania				
Igalula River Delta (IGR)	KGA3	x	x	Tanzania				
Igalula River Delta (IGR)	KGB4	x	x	Tanzania				
Igalula River Delta (IGR)	KGB5	x	x	Tanzania				
Kalambo lake (KAL)	38C3	x		Zambia	8°36'6.27"S	31°11'13.24"E	Theis et al. 2014	
Kalambo lake (KAL)	38C4	x		Zambia			Theis et al. 2014	
Kalambo lake (KAL)	38C5	x		Zambia			Theis et al. 2014	
Kalambo lake (KAL)	38C6	x		Zambia			Theis et al. 2014	
Kalambo lake (KAL)	38C7	x		Zambia			Theis et al. 2014	
Kalambo lake (KAL)	38C8	x		Zambia			Theis et al. 2014	
Kalambo lake (KAL)	38C9	x		Zambia			Theis et al. 2014	
Kalambo lake (KAL)	38D1	x		Zambia			Theis et al. 2014	
Kalambo lake (KAL)	38D2	x		Zambia			Theis et al. 2014	
Kalambo lake (KAL)	38D3	x		Zambia			Theis et al. 2014	
Kalambo lake (KAL)	38D4	x		Zambia			Theis et al. 2014	
Kalambo lake (KAL)	38D5	x		Zambia			Theis et al. 2014	
Kalambo lake (KAL)	38D7	x		Zambia			Theis et al. 2014	
Kalambo lake (KAL)	38D8	x		Zambia			Theis et al. 2014	
Kalambo lake (KAL)	38D9	x		Zambia			Theis et al. 2014	
Kalambo lake (KAL)	38E1	x		Zambia			Theis et al. 2014	
Kalambo lake (KAL)	38E2	x		Zambia			Theis et al. 2014	
Kalambo lake (KAL)	38E3	x		Zambia			Theis et al. 2014	
Kalambo lake (KAL)	38E4	x		Zambia			Theis et al. 2014	
Kalambo lake (KAL)	38E5	x		Zambia			Theis et al. 2014	
Kalambo lake (KAL)	38E6	x		Zambia			Theis et al. 2014	
Kalambo lake (KAL)	38E7	x		Zambia			Theis et al. 2014	
Kalambo lake (KAL)	38E8	x		Zambia			Theis et al. 2014	
Kalambo lake (KAL)	38F1	x		Zambia			Theis et al. 2014	
Kalambo lake (KAL)	38F2	x		Zambia			Theis et al. 2014	
Kalambo lake (KAL)	38F3	x		Zambia			Theis et al. 2014	
Kalambo lake (KAL)	38F4	x		Zambia			Theis et al. 2014	
Kalambo lake (KAL)	38F5	x		Zambia			Theis et al. 2014	
Kalambo lake (KAL)	38G6	x		Zambia			Theis et al. 2014	
Kalambo lake (KAL)	58F2		x	Zambia				Egger et al. 2017
Kalambo lake (KAL)	58H1		x	Zambia				Egger et al. 2017
Kalambo lake (KAL)	58H4		x	Zambia				Egger et al. 2017
Kalambo lake (KAL)	58H5		x	Zambia				Egger et al. 2017
Kalambo lake (KAL)	58I1		x	Zambia				Egger et al. 2017
Kalambo stream below falls (KBF)	3871	x	x	Zambia	8°35'47.24"S	31°14'20.92"E		
Kalambo stream 1 (KA1)	60A9	x		Zambia	8°35'35.23"S	31°11'6.18"E	Theis et al. 2014	
Kalambo stream 1 (KA1)	60B8	x		Zambia			Theis et al. 2014	
Kalambo stream 1 (KA1)	60F7	x		Zambia			Theis et al. 2014	
Kalambo stream 1 (KA1)	60F8	x		Zambia			Theis et al. 2014	
Kalambo stream 1 (KA1)	60F9	x	x	Zambia			Theis et al. 2014	Egger et al. 2017
Kalambo stream 1 (KA1)	60G1	x		Zambia			Theis et al. 2014	
Kalambo stream 1 (KA1)	60F6		x	Zambia				Egger et al. 2017
Kalambo stream 1 (KA1)	60A7		x	Zambia				Egger et al. 2017
Kalambo stream 1 (KA1)	60H4		x	Zambia				Egger et al. 2017
Kalambo stream 1 (KA1)	60H5		x	Zambia				Egger et al. 2017
Kalambo stream 2 (KA2)	AsBur_6	x		Zambia	8°35'6.24"S	31°12'29.32"E		
Kalambo stream 2 (KA2)	DQH3	x		Zambia			Theis et al. 2014	
Kalambo stream 2 (KA2)	DQH4	x		Zambia			Theis et al. 2014	
Kalambo stream 2 (KA2)	DQH5	x		Zambia			Theis et al. 2014	
Kalambo stream 2 (KA2)	DQH6	x	x	Zambia			Theis et al. 2014	Egger et al. 2017
Kalambo stream 2 (KA2)	DQH7	x		Zambia			Theis et al. 2014	
Kalambo stream 2 (KA2)	DQH8	x	x	Zambia			Theis et al. 2014	Egger et al. 2017
Kalambo stream 2 (KA2)	DQH9	x		Zambia			Theis et al. 2014	
Kalambo stream 2 (KA2)	DQI1	x		Zambia			Theis et al. 2014	
Kalambo stream 2 (KA2)	DPD7		x	Zambia				Egger et al. 2017
Kalambo stream 2 (KA2)	DPD8		x	Zambia				Egger et al. 2017
Kalambo stream 2 (KA2)	DPE3		x	Zambia				Egger et al. 2017
Kalambo stream 3 (KA3)	37F6	x		Zambia	8°35'41.59"S	31°14'50.32"E	Theis et al. 2014	
Kalambo stream 3 (KA3)	37F7	x		Zambia			Theis et al. 2014	
Kalambo stream 3 (KA3)	37F8	x		Zambia			Theis et al. 2014	
Kalambo stream 3 (KA3)	37F9	x		Zambia			Theis et al. 2014	
Kalambo stream 3 (KA3)	37G1	x		Zambia			Theis et al. 2014	
Kalambo stream 3 (KA3)	37G2	x		Zambia			Theis et al. 2014	
Kalambo stream 3 (KA3)	37G3	x		Zambia			Theis et al. 2014	
Kalambo stream 3 (KA3)	37G4	x		Zambia			Theis et al. 2014	
Kalambo stream 3 (KA3)	37G8	x		Zambia			Theis et al. 2014	
Kalambo stream 3 (KA3)	37G9	x		Zambia			Theis et al. 2014	
Kalambo stream 3 (KA3)	37H1	x		Zambia			Theis et al. 2014	
Kalambo stream 3 (KA3)	37H2	x		Zambia			Theis et al. 2014	
Kalambo stream 3 (KA3)	37H3	x		Zambia			Theis et al. 2014	
Kalambo stream 3 (KA3)	37H4	x		Zambia			Theis et al. 2014	
Kalambo stream 3 (KA3)	37H5	x		Zambia			Theis et al. 2014	

Kalambo stream 3 (KA3)	37H6	x		Zambia			Theis et al. 2014	
Kalambo stream 3 (KA3)	37H7	x		Zambia			Theis et al. 2014	
Kalambo stream 3 (KA3)	37H8	x		Zambia			Theis et al. 2014	
Kalambo stream 3 (KA3)	37H9	x		Zambia			Theis et al. 2014	
Kalambo stream 3 (KA3)	37I1	x		Zambia			Theis et al. 2014	
Kalambo stream 3 (KA3)	37I2	x		Zambia			Theis et al. 2014	
Kalambo stream 3 (KA3)	37I3	x		Zambia			Theis et al. 2014	
Kalambo stream 3 (KA3)	37I5	x		Zambia			Theis et al. 2014	
Kalambo stream 3 (KA3)	38A1_1	x		Zambia			Theis et al. 2014	
Kalambo stream 3 (KA3)	38A1_3	x		Zambia			Theis et al. 2014	
Kalambo stream 3 (KA3)	38A1_4	x		Zambia			Theis et al. 2014	
Kalambo stream 3 (KA3)	38A1_5	x		Zambia			Theis et al. 2014	
Kalambo stream 3 (KA3)	58A4		x	Zambia				Egger et al. 2017
Kalambo stream 3 (KA3)	58B1		x	Zambia				Egger et al. 2017
Kalambo stream 3 (KA3)	58B3		x	Zambia				Egger et al. 2017
Kalambo stream 3 (KA3)	58B6		x	Zambia				Egger et al. 2017
Kalambo stream 3 (KA3)	58B7		x	Zambia				Egger et al. 2017
Kalambo stream 4 (KAL4)	KAL4_1	x	x	Zambia	8°36'15.99''S	31°15'17.21''E	Theis et al. 2014	
Kalambo stream 4 (KAL4)	KAL4_2	x	x	Zambia			Theis et al. 2014	
Kalambo stream 4 (KAL4)	KAL4_3	x	x	Zambia			Theis et al. 2014	
Kalambo stream 4 (KAL4)	KAL4_5	x	x	Zambia			Theis et al. 2014	
Kalemie (KKA)	1_AB4861	x	x	Democratic Republic of Kongo	5°56'50.34''S	29°11'47.62''E		
Kalungula (KLU)	61A8	x		Zambia	8°48'33.39''S	31°7'49.02''E	Theis et al. 2014	
Kalungula (KLU)	61A9	x		Zambia			Theis et al. 2014	
Kalungula (KLU)	61B1	x		Zambia			Theis et al. 2014	
Kalungula (KLU)	61B2	x		Zambia			Theis et al. 2014	
Kalungula (KLU)	61B3	x		Zambia			Theis et al. 2014	
Kalungula (KLU)	61B4	x		Zambia			Theis et al. 2014	
Kalungula (KLU)	61B5	x		Zambia			Theis et al. 2014	
Kalungula (KLU)	61B6	x		Zambia			Theis et al. 2014	
Kalungula (KLU)	61B7	x		Zambia			Theis et al. 2014	
Kalungula (KLU)	61B8	x		Zambia			Theis et al. 2014	
Kalungula (KLU)	61B9	x		Zambia			Theis et al. 2014	
Kalungula (KLU)	61C1	x	x	Zambia			Theis et al. 2014	
Kalungula (KLU)	61C2	x		Zambia			Theis et al. 2014	
Kalungula (KLU)	61C3	x		Zambia			Theis et al. 2014	
Kalungula (KLU)	61C4	x		Zambia			Theis et al. 2014	
Kalungula (KLU)	61C5	x		Zambia			Theis et al. 2014	
Kalungula (KLU)	61C6	x		Zambia			Theis et al. 2014	
Kalungula (KLU)	61C7	x		Zambia			Theis et al. 2014	
Kalungula (KLU)	61C8	x		Zambia			Theis et al. 2014	
Kalungula (KLU)	61C9	x		Zambia			Theis et al. 2014	
Kalungula (KLU)	61D1	x		Zambia			Theis et al. 2014	
Kalungula (KLU)	61D2	x	x	Zambia			Theis et al. 2014	
Kalungula (KLU)	61D3	x	x	Zambia			Theis et al. 2014	
Kalungula (KLU)	61D4	x		Zambia			Theis et al. 2014	
Kalungula (KLU)	61D5	x	x	Zambia			Theis et al. 2014	
Kalungula (KLU)	61D6	x	x	Zambia			Theis et al. 2014	
Kalungula (KLU)	61D7	x		Zambia			Theis et al. 2014	
Kalungula (KLU)	61D8	x		Zambia			Theis et al. 2014	
Kigoma (KIG)	Kigoma1		x	Tanzania	4°51'41.90''S	29°37'45.08''E		
Kigoma (KIG)	Kigoma2		x	Tanzania				
Kigoma (KIG)	Kigoma3		x	Tanzania				
Kigoma (KIG)	Kigoma4		x	Tanzania				
Kigoma (KIG)	Kigoma5	x	x	Tanzania				
Lake Chila (LCZ)	DRC8	x		Zambia	8°50'8.68''S	31°22'49.44''E	Theis et al. 2014	
Lake Chila (LCZ)	DRC9	x		Zambia			Theis et al. 2014	
Lake Chila (LCZ)	DRD1	x		Zambia			Theis et al. 2014	
Lake Chila (LCZ)	DRD2	x		Zambia			Theis et al. 2014	
Lake Chila (LCZ)	DRD3	x		Zambia			Theis et al. 2014	
Lake Chila (LCZ)	DRD4	x		Zambia			Theis et al. 2014	
Lake Chila (LCZ)	DRD5	x		Zambia			Theis et al. 2014	
Lake Chila (LCZ)	DRF3	x		Zambia			Theis et al. 2014	
Lake Chila (LCZ)	EFF3	x	x	Zambia			Theis et al. 2014	
Lake Chila (LCZ)	EFF4	x	x	Zambia			Theis et al. 2014	
Lake Chila (LCZ)	EFF5	x	x	Zambia			Theis et al. 2014	
Lake Chila (LCZ)	EFF6	x	x	Zambia			Theis et al. 2014	
Lake Chila (LCZ)	EFF7	x	x	Zambia			Theis et al. 2014	
Lake Chila (LCZ)	EFF8	x		Zambia			Theis et al. 2014	
Lake Cohoha (LCB)	IGG9	x		Burundi	2°30'47.14''S	30° 6'3.17''E		
Lake Cohoha (LCB)	IGH1	x		Burundi				
Lake Cohoha (LCB)	IGH2	x		Burundi				
Lake Cohoha (LCB)	IGH3	x		Burundi				
Lake Cohoha (LCB)	IGH4	x		Burundi				
Lake Cohoha (LCB)	IGH5	x		Burundi				
Lake Cohoha (LCB)	IGH7	x		Burundi				
Lake Cohoha (LCB)	IGH8	x		Burundi				
Lake Cohoha (LCB)	KZI2		x	Burundi				
Lake Cohoha (LCB)	LAB7		x	Burundi				
Lake Cohoha (LCB)	LAC2		x	Burundi				
Lake Cohoha (LCB)	LAC7		x	Burundi				
Lake Cohoha (LCB)	LAF8		x	Burundi				
Lake Cohoha (LCB)	AY226785	x		Burundi			Verheyen et al. 2004	
Lake Cohoha (LCB)	AY226786	x		Burundi			Verheyen et al. 2004	
Loasi (LOA)	DCD8	x		Tanzania	8°18'49.55''S	31°2'57.54''E	Theis et al. 2014	
Loasi (LOA)	DCD9	x		Tanzania			Theis et al. 2014	

Loasi (LOA)	DCE2	x		Tanzania			Theis et al. 2014	
Loasi (LOA)	DCE3	x		Tanzania			Theis et al. 2014	
Loasi (LOA)	DCE4	x		Tanzania			Theis et al. 2014	
Loasi (LOA)	DCE5	x	x	Tanzania			Theis et al. 2014	
Loasi (LOA)	DCE7	x		Tanzania			Theis et al. 2014	
Loasi (LOA)	DCE6		x	Tanzania				
Loasi (LOA)	DCF8		x	Tanzania				
Loasi (LOA)	DCG4		x	Tanzania				
Loasi (LOA)	DCG5		x	Tanzania				
Lufubu lake (LFL)	71A2	x		Zambia	8°33'36.56"S	30°43'33.79"E	Theis et al. 2014	
Lufubu lake (LFL)	71A3	x	x	Zambia			Theis et al. 2014	Egger et al. 2017
Lufubu lake (LFL)	71A4	x	x	Zambia			Theis et al. 2014	Egger et al. 2017
Lufubu lake (LFL)	71A5	x	x	Zambia			Theis et al. 2014	Egger et al. 2017
Lufubu lake (LFL)	71A6	x		Zambia			Theis et al. 2014	
Lufubu lake (LFL)	71A7	x		Zambia			Theis et al. 2014	
Lufubu lake (LFL)	71A8	x	x	Zambia			Theis et al. 2014	Egger et al. 2017
Lufubu lake (LFL)	71A9	x		Zambia			Theis et al. 2014	
Lufubu lake (LFL)	71B1	x		Zambia			Theis et al. 2014	
Lufubu lake (LFL)	AsBur_7	x		Zambia				
Lufubu lake (LFL)	71B2		x	Zambia			Theis et al. 2014	Egger et al. 2017
Lufubu stream 1 (LF1)	71D4	x		Zambia	8°35'49.31"S	30°43'38.96"E	Theis et al. 2014	
Lufubu stream 1 (LF1)	71D6	x		Zambia			Theis et al. 2014	
Lufubu stream 1 (LF1)	71D7	x		Zambia			Theis et al. 2014	
Lufubu stream 1 (LF1)	71D8	x		Zambia			Theis et al. 2014	
Lufubu stream 1 (LF1)	71D9	x		Zambia			Theis et al. 2014	
Lufubu stream 1 (LF1)	71E1	x		Zambia			Theis et al. 2014	
Lufubu stream 1 (LF1)	71E2	x		Zambia			Theis et al. 2014	
Lufubu stream 1 (LF1)	71E3	x		Zambia			Theis et al. 2014	
Lufubu stream 1 (LF1)	71E4	x		Zambia			Theis et al. 2014	
Lufubu stream 1 (LF1)	71E5	x		Zambia			Theis et al. 2014	
Lufubu stream 2 (LF2)	FGA2	x		Zambia	8°41'9.37"S	30°33'51.90"E	Theis et al. 2014	
Lufubu stream 2 (LF2)	FGA3	x	x	Zambia			Theis et al. 2014	Egger et al. 2017
Lufubu stream 2 (LF2)	FGA4	x	x	Zambia			Theis et al. 2014	Egger et al. 2017
Lufubu stream 2 (LF2)	FGA5	x		Zambia			Theis et al. 2014	
Lufubu stream 2 (LF2)	FGA7	x		Zambia			Theis et al. 2014	
Lufubu stream 2 (LF2)	FGA8	x	x	Zambia			Theis et al. 2014	Egger et al. 2017
Lufubu stream 2 (LF2)	FGB1	x		Zambia			Theis et al. 2014	
Lufubu stream 2 (LF2)	FGB2	x		Zambia			Theis et al. 2014	
Lufubu stream 2 (LF2)	FGB8	x		Zambia			Theis et al. 2014	
Lufubu stream 2 (LF2)	FGE9	x		Zambia			Theis et al. 2014	
Lufubu stream 2 (LF2)	FGF4	x		Zambia			Theis et al. 2014	
Lufubu stream 2 (LF2)	FGF9	x		Zambia			Theis et al. 2014	
Lufubu stream 2 (LF2)	FGG1	x		Zambia			Theis et al. 2014	
Lufubu stream 2 (LF2)	FGE7		x	Zambia				Egger et al. 2017
Lufubu stream 2 (LF2)	FGF1		x	Zambia				Egger et al. 2017
Lunzua lake (LzL)	59C8	x		Zambia	8°44'57.13"S	31°10'21.86"E	Theis et al. 2014	
Lunzua lake (LzL)	59C9	x	x	Zambia			Theis et al. 2014	Egger et al. 2017
Lunzua lake (LzL)	59D1	x		Zambia			Theis et al. 2014	
Lunzua lake (LzL)	59D2	x		Zambia			Theis et al. 2014	
Lunzua lake (LzL)	59D3	x		Zambia			Theis et al. 2014	
Lunzua lake (LzL)	59D4	x		Zambia			Theis et al. 2014	
Lunzua lake (LzL)	59D5	x		Zambia			Theis et al. 2014	
Lunzua lake (LzL)	59D6	x		Zambia			Theis et al. 2014	
Lunzua lake (LzL)	59D7	x		Zambia			Theis et al. 2014	
Lunzua lake (LzL)	59D8	x		Zambia			Theis et al. 2014	
Lunzua lake (LzL)	59D9	x		Zambia			Theis et al. 2014	
Lunzua lake (LzL)	59E1	x	x	Zambia			Theis et al. 2014	Egger et al. 2017
Lunzua lake (LzL)	59E2	x		Zambia			Theis et al. 2014	
Lunzua lake (LzL)	59E3	x		Zambia			Theis et al. 2014	
Lunzua lake (LzL)	59E4	x		Zambia			Theis et al. 2014	
Lunzua lake (LzL)	59E5	x		Zambia			Theis et al. 2014	
Lunzua lake (LzL)	59E6	x		Zambia			Theis et al. 2014	
Lunzua lake (LzL)	59E7	x		Zambia			Theis et al. 2014	
Lunzua lake (LzL)	59E8	x		Zambia			Theis et al. 2014	
Lunzua lake (LzL)	59E9	x	x	Zambia			Theis et al. 2014	Egger et al. 2017
Lunzua lake (LzL)	59F1	x	x	Zambia			Theis et al. 2014	Egger et al. 2017
Lunzua lake (LzL)	59F2	x		Zambia			Theis et al. 2014	
Lunzua lake (LzL)	59F3	x		Zambia			Theis et al. 2014	
Lunzua lake (LzL)	59F4	x		Zambia			Theis et al. 2014	
Lunzua lake (LzL)	59C6		x	Zambia				Egger et al. 2017
Lunzua stream 1 (Lz1)	62I6	x		Zambia	8°47'23.51"S	31°8'14.33"E	Theis et al. 2014	
Lunzua stream 1 (Lz1)	65C2		x	Zambia				Egger et al. 2017
Lunzua stream 1 (Lz1)	65D9		x	Zambia				Egger et al. 2017
Lunzua stream 1 (Lz1)	62I7	x		Zambia			Theis et al. 2014	
Lunzua stream 1 (Lz1)	62I8	x	x	Zambia			Theis et al. 2014	Egger et al. 2017
Lunzua stream 1 (Lz1)	62I9	x		Zambia			Theis et al. 2014	
Lunzua stream 1 (Lz1)	65A2	x		Zambia			Theis et al. 2014	
Lunzua stream 1 (Lz1)	65A5	x		Zambia			Theis et al. 2014	
Lunzua stream 1 (Lz1)	65A7	x		Zambia			Theis et al. 2014	
Lunzua stream 1 (Lz1)	65D1		x	Zambia				Egger et al. 2017
Lunzua stream 1 (Lz1)	65B4		x	Zambia				Egger et al. 2017
Malagarasi (MAL)	KGD8	x	x	Tanzania	29° 50' 32" E	05° 12' 43" S		
Malagarasi (MAL)	KGD9	x	x	Tanzania				
Malagarasi (MAL)	KGE1	x	x	Tanzania				
Malagarasi (MAL)	KGE2	x	x	Tanzania				
Malagarasi (MAL)	KGE3	x	x	Tanzania				

Muzi (MUZ)	DCA1	x		Tanzania	8°23'1.84"S	31°7'47.15"E	Theis et al. 2014	
Muzi (MUZ)	DCA3	x		Tanzania			Theis et al. 2014	
Muzi (MUZ)	DCA4	x		Tanzania			Theis et al. 2014	
Muzi (MUZ)	DCA5	x		Tanzania			Theis et al. 2014	
Muzi (MUZ)	DCA6	x		Tanzania			Theis et al. 2014	
Muzi (MUZ)	DCA7	x		Tanzania			Theis et al. 2014	
Muzi (MUZ)	DCA8	x		Tanzania			Theis et al. 2014	
Muzi (MUZ)	DCA9	x		Tanzania			Theis et al. 2014	
Muzi (MUZ)	DCB1	x		Tanzania			Theis et al. 2014	
Muzi (MUZ)	DCC8		x	Tanzania				
Ndole Bay (NDB)	FNA4	x		Zambia	8°28'34.61"S	30°26'57.48"E	Theis et al. 2014	
Ndole Bay (NDB)	FNA5	x		Zambia			Theis et al. 2014	
Ndole Bay (NDB)	FNA6	x		Zambia			Theis et al. 2014	
Ndole Bay (NDB)	FNA7	x		Zambia			Theis et al. 2014	
Ndole Bay (NDB)	FNA8	x		Zambia			Theis et al. 2014	
Ndole Bay (NDB)	FND6	x		Zambia			Theis et al. 2014	
Ndole Bay (NDB)	FND7	x		Zambia			Theis et al. 2014	
Ndole Bay (NDB)	FND8	x		Zambia			Theis et al. 2014	
Ndole Bay (NDB)	FND9	x		Zambia			Theis et al. 2014	
Ndole Bay (NDB)	FNE1	x		Zambia			Theis et al. 2014	
Ndole Bay (NDB)	FNE2	x		Zambia			Theis et al. 2014	
Ndole Bay (NDB)	FNE3	x		Zambia			Theis et al. 2014	
Ndole Bay (NDB)	FNE4	x		Zambia			Theis et al. 2014	
Ndole Bay (NDB)	FNA9		x	Zambia				
Ndole Bay (NDB)	FNB1		x	Zambia				
Ndole Bay (NDB)	FNH2		x	Zambia				
Ndole Bay (NDB)	FNB4		x	Zambia				
Ndole Bay (NDB)	FNI3		x	Zambia				
Ninde (NIN)	DCD1	x	x	Tanzania	7°40'51.10"S	30° 43'20.63"E	Theis et al. 2014	
Ninde (NIN)	DCD2	x		Tanzania			Theis et al. 2014	
Ninde (NIN)	DCD3	x	x	Tanzania			Theis et al. 2014	
Ninde (NIN)	DCD4	x	x	Tanzania			Theis et al. 2014	
Ninde (NIN)	DCD5	x	x	Tanzania			Theis et al. 2014	
Ninde (NIN)	DCD6	x		Tanzania			Theis et al. 2014	
Ninde (NIN)	DCD7	x	x	Tanzania			Theis et al. 2014	
Ruzizi River (RUR)	IGE8	x		Burundi	3°20'15.48"S	29°16'25.75"E		
Ruzizi River (RUR)	KBG7	x	x	Burundi				
Ruzizi River (RUR)	KBC6	x	x	Burundi				
Ruzizi River (RUR)	KBC3	x	x	Burundi				
Ruzizi River (RUR)	KBC8	x	x	Burundi				
Ruzizi River (RUR)	KBG6	x	x	Burundi				
Salzburger Lab (LAB)	AsBur_10	x						
Salzburger Lab (LAB)	AsBur_11	x						
Salzburger Lab (LAB)	AY930000	x					Salzburger et al. (2005)	
Salzburger Lab (LAB)	AY930001	x					Salzburger et al. (2005)	
Salzburger Lab (LAB)	AB_Lab3		x					
Salzburger Lab (LAB)	AB_Lab4		x					
Salzburger Lab (LAB)	AB_Lab5		x					
Salzburger Lab (LAB)	AB_Lab1		x					
Salzburger Lab (LAB)	AB_Lab2		x					
Sebele (SEB)	AsBur_1	x		Democratic Republic of Kongo	4°18'32.4"S	29°03'42.84"E		
Sebele (SEB)	Kongo_Se_1	x	x	Democratic Republic of Kongo				
Sebele (SEB)	Kongo_Se_2	x	x	Democratic Republic of Kongo				
Sebele (SEB)	Kongo_Se_3	x	x	Democratic Republic of Kongo				
Sebele (SEB)	Kongo_Se_4	x	x	Democratic Republic of Kongo				
Sebele (SEB)	Kongo_Se_5	x	x	Democratic Republic of Kongo				
Sumba (SUM)	1825	x		Zambia	8°40'18.58"S	31°11'33.94"E	Theis et al. 2014	
Sumba (SUM)	1826	x		Zambia			Theis et al. 2014	
Sumba (SUM)	1827	x		Zambia			Theis et al. 2014	
Sumba (SUM)	1828	x		Zambia			Theis et al. 2014	
Sumba (SUM)	1829	x		Zambia			Theis et al. 2014	
Sumba (SUM)	1830	x		Zambia			Theis et al. 2014	
Sumba (SUM)	1831	x		Zambia			Theis et al. 2014	
Sumba (SUM)	1832	x		Zambia			Theis et al. 2014	
Sumba (SUM)	1834	x		Zambia			Theis et al. 2014	
Toby's (TOB)	38A6	x		Zambia	8°37'25.99"S	31°12'2.86"E	Theis et al. 2014	
Toby's (TOB)	38A7	x		Zambia			Theis et al. 2014	
Toby's (TOB)	38A8	x		Zambia			Theis et al. 2014	
Toby's (TOB)	38A9	x		Zambia			Theis et al. 2014	
Toby's (TOB)	38B1	x		Zambia			Theis et al. 2014	
Toby's (TOB)	38B2	x		Zambia			Theis et al. 2014	
Toby's (TOB)	38B3	x		Zambia			Theis et al. 2014	
Toby's (TOB)	38B4	x		Zambia			Theis et al. 2014	
Toby's (TOB)	38B5	x		Zambia			Theis et al. 2014	
Toby's (TOB)	38B6	x		Zambia			Theis et al. 2014	
Toby's (TOB)	38B7	x		Zambia			Theis et al. 2014	
Toby's (TOB)	38C1	x		Zambia			Theis et al. 2014	
Toby's (TOB)	38C2	x		Zambia			Theis et al. 2014	
Toby's (TOB)	38H1	x		Zambia			Theis et al. 2014	
Toby's (TOB)	38H2	x		Zambia			Theis et al. 2014	
Toby's (TOB)	38H3	x		Zambia			Theis et al. 2014	
Toby's (TOB)	38H4	x		Zambia			Theis et al. 2014	
Toby's (TOB)	38H5	x		Zambia			Theis et al. 2014	
Toby's (TOB)	38H6	x		Zambia			Theis et al. 2014	
Toby's (TOB)	38H8	x		Zambia			Theis et al. 2014	
Toby's (TOB)	38H9	x		Zambia			Theis et al. 2014	

Toby's (TOB)	38I1	x		Zambia			Theis et al. 2014	
Toby's (TOB)	38I2	x		Zambia			Theis et al. 2014	
Toby's (TOB)	38I3	x		Zambia			Theis et al. 2014	
Toby's (TOB)	38I5	x		Zambia			Theis et al. 2014	
Toby's (TOB)	38I6	x		Zambia			Theis et al. 2014	
Toby's (TOB)	38I7	x		Zambia			Theis et al. 2014	
Toby's (TOB)	38I8	x		Zambia			Theis et al. 2014	
Toby's (TOB)	38I9	x		Zambia			Theis et al. 2014	
Toby's (TOB)	46F1	x		Zambia			Theis et al. 2014	
Wonzye (WON)	46G4	x		Zambia	8°45'1.76"S	31°7'49.02"E	Theis et al. 2014	
Wonzye (WON)	46G5	x		Zambia			Theis et al. 2014	
Wonzye (WON)	46G6	x		Zambia			Theis et al. 2014	
Wonzye (WON)	46G7	x		Zambia			Theis et al. 2014	
Wonzye (WON)	46G8	x		Zambia			Theis et al. 2014	
Wonzye (WON)	46G9	x		Zambia			Theis et al. 2014	
Wonzye (WON)	46H1	x		Zambia			Theis et al. 2014	
Wonzye (WON)	46H2	x		Zambia			Theis et al. 2014	
Wonzye (WON)	46H4	x		Zambia			Theis et al. 2014	
Wonzye (WON)	46H5	x		Zambia			Theis et al. 2014	
Wonzye (WON)	46H6	x		Zambia			Theis et al. 2014	
Wonzye (WON)	46H7	x		Zambia			Theis et al. 2014	
Wonzye (WON)	46H8	x		Zambia			Theis et al. 2014	
Wonzye (WON)	46H9	x		Zambia			Theis et al. 2014	
Wonzye (WON)	46I1	x		Zambia			Theis et al. 2014	
Wonzye (WON)	46I3	x		Zambia			Theis et al. 2014	
Wonzye (WON)	46I4	x		Zambia			Theis et al. 2014	
Wonzye (WON)	46I5	x		Zambia			Theis et al. 2014	
Wonzye (WON)	46I6	x		Zambia			Theis et al. 2014	
Wonzye (WON)	46I7	x		Zambia			Theis et al. 2014	
Wonzye (WON)	46I8	x		Zambia			Theis et al. 2014	
Wonzye (WON)	46I9	x		Zambia			Theis et al. 2014	
Wonzye (WON)	47D9	x		Zambia			Theis et al. 2014	
Wonzye (WON)	47E1	x		Zambia			Theis et al. 2014	
Wonzye (WON)	47E2	x		Zambia			Theis et al. 2014	
Wonzye (WON)	47E3	x		Zambia			Theis et al. 2014	
Wonzye (WON)	47E4	x		Zambia			Theis et al. 2014	
Wonzye (WON)	47E5	x		Zambia			Theis et al. 2014	
Wonzye (WON)	47E7	x		Zambia			Theis et al. 2014	
Wonzye (WON)	47E8	x		Zambia			Theis et al. 2014	
Wonzye (WON)	54A1	x		Zambia			Theis et al. 2014	
Wonzye (WON)	54A2	x		Zambia			Theis et al. 2014	
Wonzye (WON)	54A3	x		Zambia			Theis et al. 2014	
Wonzye (WON)	54A4	x		Zambia			Theis et al. 2014	
Wonzye (WON)	54A5	x		Zambia			Theis et al. 2014	
Wonzye (WON)	54A6	x		Zambia			Theis et al. 2014	
Wonzye (WON)	54A7	x		Zambia			Theis et al. 2014	
Wonzye (WON)	54A8	x		Zambia			Theis et al. 2014	
Wonzye (WON)	54A9	x		Zambia			Theis et al. 2014	
Wonzye (WON)	DRF4	x		Zambia			Theis et al. 2014	
Wonzye (WON)	DRF5	x	x	Zambia			Theis et al. 2014	
Wonzye (WON)	DRF6	x	x	Zambia			Theis et al. 2014	
Wonzye (WON)	DRF7	x		Zambia			Theis et al. 2014	
Wonzye (WON)	DRF8	x		Zambia			Theis et al. 2014	
Wonzye (WON)	DRF9	x		Zambia			Theis et al. 2014	
Wonzye (WON)	DRG1	x	x	Zambia			Theis et al. 2014	
Wonzye (WON)	DRG2	x	x	Zambia			Theis et al. 2014	
Wonzye (WON)	DRG3	x		Zambia			Theis et al. 2014	
Wonzye (WON)	DRG4	x		Zambia			Theis et al. 2014	
