# Supplementary material for: The puzzling phylogeography of the haplochromine cichlid fish Astatotilapia burtoni
Source: Ecol Evol. 2018 May 2;8(11):5637–48. doi: 10.1002/ece3.4092 (PMC6010872; doi:10.1002/ece3.4092)
Supplement: Supplementary file 3 [file ECE3-8-5637-s003.pdf]

Species/population	Sample ID	Reads total	Unique aligned	Coverage
**Astatotilapia calliptera**	A.cal2	3634515	2907540	56.20
**Astatotilapia flavijosephi**	LJD2	4413633	3661190	73.81
**Haplochromis paludinosus**	KYG1	4373902	3491342	67.09
**CHL**	66G7	4306634	3393991	59.13
	66G9	5408609	4307174	73.71
	66H2	3793070	3004842	53.40
	66H7	3600897	2854866	50.52
	BJE5	4814407	3691674	55.78
**CH1**	BJA2	5517534	4355118	74.76
	BJA4	5216670	4133274	71.71
	BJC2	6936405	5215942	75.38
	BJC9	5730734	4396451	78.50
	BJD1	4155384	4041726	72.43
**CRO**	MIB3	5471588	4638655	84.41
	MIB4	6475763	5467179	98.04
	MIB5	4936685	4163616	76.07
	MIB6	4316327	3604514	62.46
	MIC2	6315285	5330279	95.71
**FID**	50H3	4981327	4151920	69.67
	50H4	3358637	2842851	49.94
	50H5	2370904	1926962	35.13
	50H6	3783350	3121273	51.99
	50I3	7930588	6659189	116.62
**HHL**	HHAB_1	5202670	4456486	83.01
	HHAB_2	4524205	3882232	69.74
	HHAB_3	6210892	5325259	93.42
	HHAB_4	1225434	1056648	25.83
	HHAB_5	4006812	3432413	62.86
**HHW**	HHAB_6	3984772	3342895	63.37
	HHAB_7	5495297	4654082	82.86
	HHAB_8	4647000	3900913	74.61
	HHAB_9	3694607	3117218	56.26
	HHAB_10	3741413	3151699	57.71
**IGR**	KGA1	1710651	1455677	27.40
	KGA2	2643093	2261034	41.57
	KGA3	1568293	1346563	27.08
	KGB4	3075569	2637439	48.38
	KGB5	3232369	2767223	50.02
**KA1**	60A7	7090057	5581004	91.35
	60F6	7496198	5903696	96.67
	60F9	6353965	4960770	89.74
	60H4	7132296	5511818	89.75
	60H5	8368165	6261782	104.77
**KA2**	DQH6	6807679	5412520	91.37
	DQH8	5976501	4666598	84.88
	DPD7	7668848	5802614	86.31
	DPD8	8576237	6501831	97.31
	DPE3	8419881	6325941	109.86
**KA3**	58A4	5890497	4673735	77.76
	58B1	4725954	3744388	63.95
	58B3	5385760	4257885	72.89
	58B7	6939948	5507973	92.88
	58B6	5399088	4229358	78.16
**KBF**	3871	4220211	3524951	61.96

**KA4**	KAL4_1	566924	477534	12.98
	KAL4_2	553388	465169	12.98
	KAL4_3	3681339	3104522	55.84
	KAL4_5	2375589	1999924	36.62
**KAL**	58F2	4583729	4583729	72.67
	58H1	6231302	4739617	71.80
	58H4	5928364	4499429	77.85
	58H5	6292836	4728277	80.43
	58I1	6060088	4448807	72.08
**KIG**	Kigoma1	470370	403336	13.87
	Kigoma2	563449	481482	15.10
	Kigoma3	240568	206683	11.46
	Kigoma4	825649	707537	15.59
	Kigoma5	555424	475957	12.56
**KKA**	1_AB4861	1374950	1155291	22.56
**KLU**	61C1	11697043	9822728	166.62
	61D2	11399763	9601808	164.64
	61D3	6910164	5776859	92.28
	61D5	5363604	4481116	73.02
	61D6	5067731	4211442	71.31
**LAB**	AB_Lab3	7060946	6022192	106.86
	AB_Lab4	2456216	2103358	41.73
	AB_Lab5	817689	700875	16.23
	AB_Lab1	4722954	4054569	73.15
	AB_Lab2	5501968	4716095	81.85
**LCB**	KZI2	1029263	876023	18.92
	LAB7	509019	429304	11.98
	LAC2	4799084	4085767	71.76
	LAC7	4486585	3798159	64.83
	LAF8	4531258	3851330	86.51
**LCZ**	EFF3	7436130	6275878	108.99
	EFF4	8602604	7262823	126.46
	EFF5	5679234	4811420	84.38
	EFF6	5836239	4940282	85.58
	EFF7	6905069	5818697	99.55
**LF2**	FGA3	5305502	4355434	79.19
	FGA4	6347419	5083918	92.43
	FGA8	5274045	4298075	78.15
	FGE7	5982541	4892455	88.95
	FGF1	6377010	5218061	94.87
**LFL**	71A3	6650459	5396054	98.11
	71A4	6171942	5016950	91.22
	71A5	5157020	4231417	76.93
	71A8	5295057	4319980	78.55
	71B2	5853944	4776764	86.85
**LOA**	DCE5	7074087	5912129	100.90
	DCE6	7503752	6230620	106.18
	DCF8	9153576	7597143	126.76
	DCG4	5298452	4433637	75.98
	DCG5	5841271	4864689	81.92
**LZ1**	62I8	6074242	4729764	78.82
	65D1	6077695	4704172	84.31
	65B4	7295222	5444282	92.18
	65C2	7165262	5278389	91.41
	65D9	6650205	4986219	84.15

**LZL**	59C2	7143945	5699675	95.44
	59E1	9114962	7734463	134.48
	59E9	8385540	6356436	109.67
	59F1	7618768	6148951	106.32
	59C6	6399129	5014776	90.90
**MAL**	KGD8	4101666	3504386	62.90
	KGD9	4548167	3883890	70.12
	KGE1	4984273	4272860	77.03
	KGE2	4917262	4216782	75.99
	KGE3	5299232	4533580	81.37
**MUZ**	DCC8	4631123	3803543	57.88
**NDB**	FNA9	7733341	6491694	112.43
	FNB1	9275533	7766163	136.67
	FNH2	7625357	6364091	110.04
	FNB4	4751199	3951301	64.01
	FNI3	5891735	4958873	86.51
**NIN**	DCD1	6805830	5692141	96.77
	DCD3	7866602	6569194	112.68
	DCD4	5167183	4358039	77.76
	DCD5	5422480	4525654	78.58
	DCD7	6135391	5159723	90.83
**RUL**	KBH9	841778	717856	16.13
	KBI2	973105	832586	18.16
	KBI3	864648	738765	16.67
	KZB4	3431411	2931910	52.03
	KBI9	4175751	3570531	62.38
**RUR**	KBC3	1077895	925888	18.99
	KBC6	873599	746830	16.87
	KBC8	3633437	3092833	54.79
	KBG6	2375589	1999924	57.04
	KBG7	4383737	3746487	65.05
**SEB**	Kongo_Se_1	4233923	3644366	65.59
	Kongo_Se_2	4111891	3539086	62.70
	Kongo_Se_3	8186830	7009369	121.06
	Kongo_Se_4	8249259	7101411	123.42
	Kongo_Se_5	9547690	8188871	143.03
**WON**	DRF5	833475	704563	16.40
	DRF6	767200	642277	15.04
	DRG1	787800	658020	15.59
	DRG2	2401311	2010648	35.69
	DRG5	4821278	4046427	71.06
